# Delayed Diagnosis of Suspected Osteogenesis Imperfecta in a Young Adult with Recurrent Low-Energy Fractures: A Case Report

**DOI:** 10.7759/cureus.106351

**Published:** 2026-04-03

**Authors:** Salma Drari, Zineb Baba, Ahmed Mougui, Imane El Bouchti

**Affiliations:** 1 Rheumatology, Hospital Arrazi, Centre Hospitalier Universitaire (CHU) Mohammed VI, Faculté de Médecine et de Pharmacie de Marrakech (FMPM), Université Cadi Ayyad (UCA), Marrakech, MAR

**Keywords:** biphosphonate therapy, bone fragility, genetic bone disorder, osteogenesis imperfecta, young adult

## Abstract

Osteogenesis imperfecta (OI) is a rare heritable connective tissue disorder characterized by bone fragility and heterogeneous clinical presentation. Mild phenotypes may remain undiagnosed for prolonged periods, particularly when clinical manifestations are subtle or overlooked.

We report the case of a 19-year-old man with a history of recurrent low-energy fractures since the age of eight. The patient sustained multiple fractures involving long bones, associated with progressive deformities of the lower extremities. Notably, no etiological workup had been performed during childhood. On admission, physical examination revealed blue sclerae. A positive family history was identified, with recurrent fractures reported in two maternal uncles. Based on the clinical presentation and family history, a diagnosis of type I osteogenesis imperfecta was established.

This case underscores the critical importance of early recognition of suggestive clinical features in children presenting with recurrent fractures. Improved clinician awareness may help reduce diagnostic delays, prevent complications, and enable timely genetic counseling and appropriate multidisciplinary management.

## Introduction

Osteogenesis imperfecta (OI) is an inherited connective tissue disorder characterized by bone fragility and an increased susceptibility to fractures. It is most commonly caused by mutations affecting type I collagen, particularly in the *COL1A1* and *COL1A2* genes [1,2]. Clinical severity varies widely, ranging from perinatal lethal forms to mild phenotypes presenting with recurrent fractures during childhood. This variability is reflected in the Sillence classification, which categorizes OI into different types based on clinical and radiological features [3].

In moderate forms, diagnosis is often delayed, particularly when characteristic clinical features go unrecognized. Early identification is essential to prevent complications and to ensure appropriate management, including genetic counseling. However, in resource-limited settings, the diagnosis may rely primarily on clinical and radiological findings in the absence of molecular confirmation.

We report the case of a young adult with a clinical presentation highly suggestive of osteogenesis imperfecta, in whom the diagnosis was established late following recurrent low-energy fractures beginning in childhood.

## Case presentation

A 19-year-old man was referred to our department for the evaluation of recurrent fractures evolving since childhood. He reported multiple long-bone fractures following low-energy trauma, beginning at the age of eight years. Overall, the patient sustained at least three documented long-bone fractures over an 11-year period, corresponding to an estimated frequency of approximately 0.3 fractures per year.

**Figure 1:**
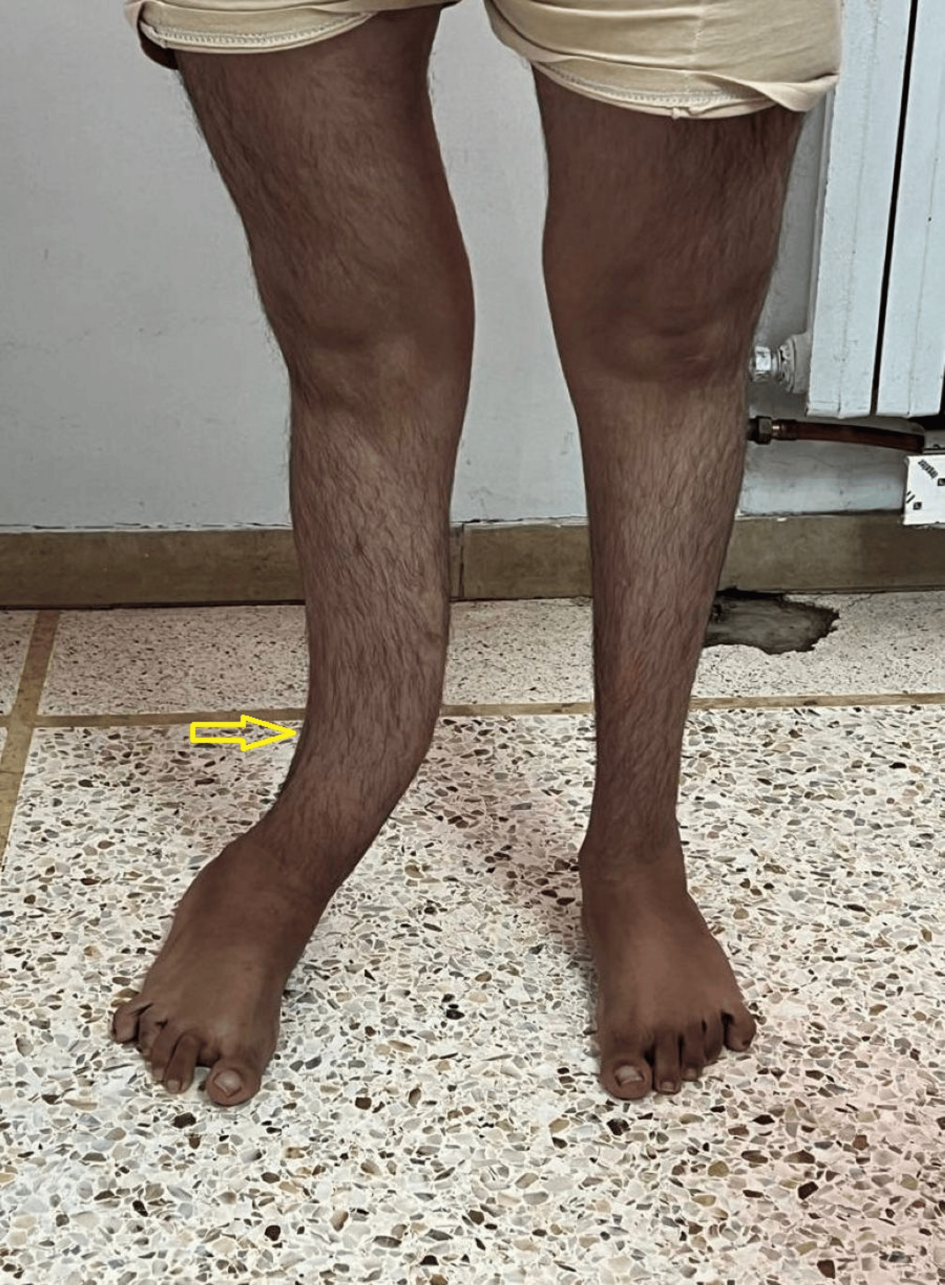
Clinical photograph showing bilateral lower limb deformities with tibial bowing (yellow arrow), in the context of osteogenesis imperfecta.

The first fracture involved the metaphysis of the right femur at the age of eight and was managed with cast immobilization. At the age of ten, he sustained a second fracture involving the distal part of the right leg after a fall from standing height. Initial orthopedic management resulted in transient improvement. However, recurrent fractures at the same site led to progressive deformity of the right lower limb. At the age of fifteen, he presented with a third fracture of the left femoral metaphysis requiring surgical fixation with osteosynthesis (Table 1 and Figure 1).

**Table 1:** Fracture history

Age	Bone	Mechanism	Treatment
8 years	Right femur	Low-energy trauma	Cast
10 years	Right leg	Fall from standing height	Orthopedic
15 years	Left femur	Low-energy trauma	Surgery

Despite recurrent fractures over a 11-year period, no etiological workup was performed during childhood. Information regarding fracture healing time was not available.

**Figure 2:**
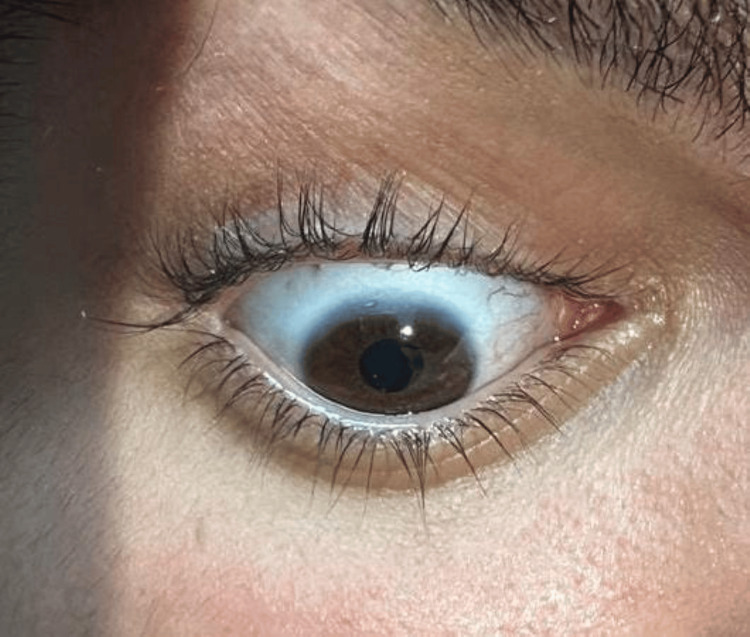
Blue sclera in the setting of osteogenesis imperfecta

On examination, the patient’s height was 159 cm and weight 71 kg, which is slightly below average for age. Blue sclerae were clearly visible (Figure 2). There was no evidence of dentinogenesis imperfecta, hearing impairment, or joint hyperlaxity. Family history revealed recurrent fractures in two maternal uncles.

**Figure 3:**
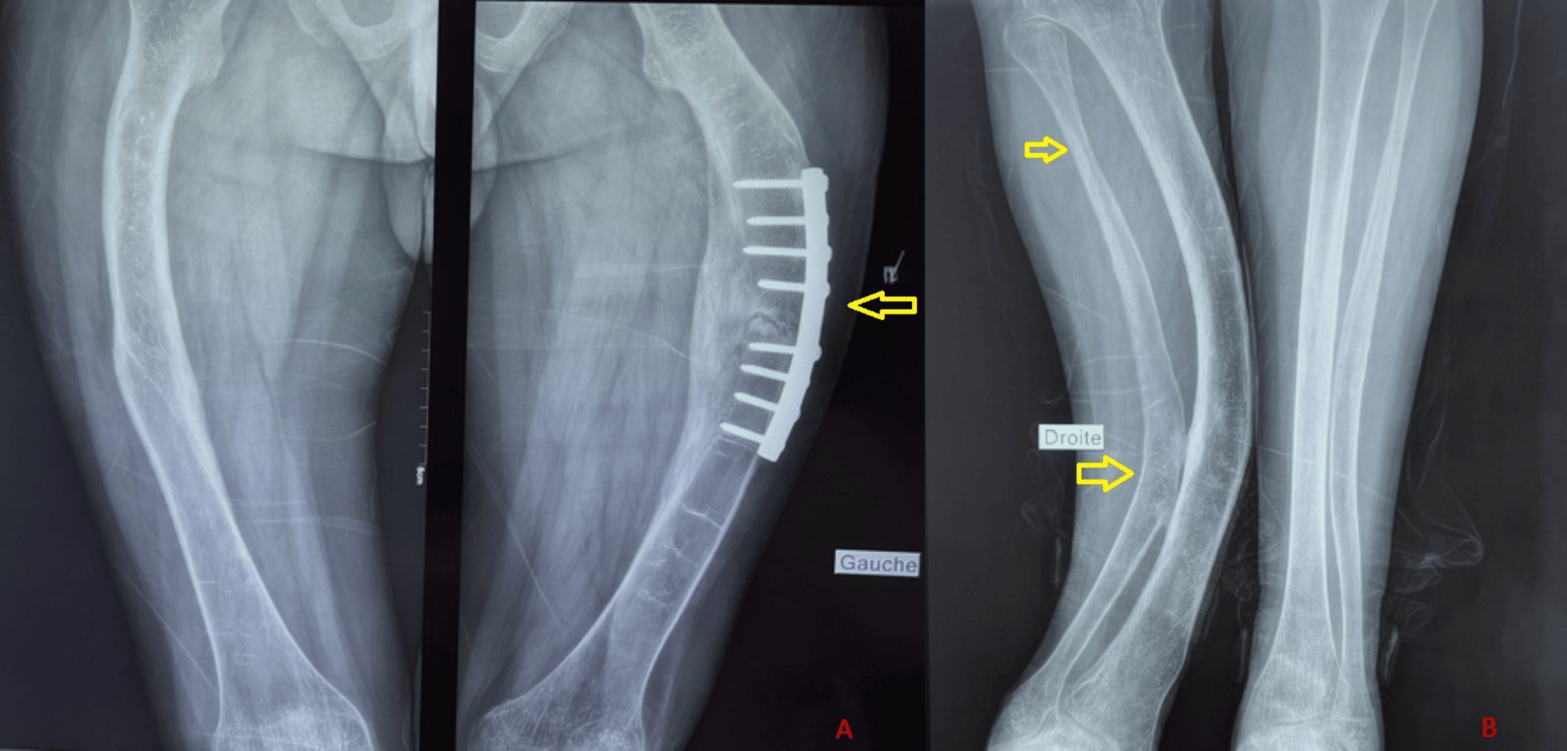
Anteroposterior radiograph of the lower limbs demonstrating: (A) Long-bone deformity with osteosynthesis hardware (arrow), associated with bowing. (B) Marked deformity of the long bones with cortical thinning (arrows), consistent with skeletal fragility suggestive of osteogenesis imperfecta.

Radiographic evaluation showed sequelae of previous fractures associated with bone deformities and cortical thinning (Figure 3). Dual-energy X-ray absorptiometry (DXA) revealed significantly reduced bone mineral density consistent with osteoporosis (Z-scores: −4.4 at the femoral neck, −3.9 at the lumbar spine, and −3.6 at the forearm).

A comprehensive laboratory workup was performed prior to bisphosphonate therapy. No inflammatory syndrome was detected. Serum calcium was 89.32 mg/L with albumin at 47.27 g/L, phosphate at 44.52 mg/L, 25-hydroxyvitamin D at 11.7 ng/mL, and parathyroid hormone (PTH) at 119 pg/mL. Alkaline phosphatase was 150 IU/L. Renal function was preserved, with a creatinine level of 5.54 mg/L (Table 2).

**Table 2:** Laboratory test findings with reference ranges

	Patient Result	Reference Range
C-reactive protein (CRP)	2.60 mg/L	0-5 mg/L
Serum calcium	89.32 mg/L	85–105 mg/L
Albumin	47.27 g/L	32-45 g/L
Phosphate	44.52 mg/L	25-45 mg/L
25-Hydroxyvitamin D	11.7 ng/mL	30-100 ng/mL
Parathyroid hormone (PTH)	119 pg/mL	15-65 pg/mL
Alkaline phosphatase (PAL)	150 UI/L	40-150 UI/L
Creatinine	5.54 mg/L	7-12 mg/L
Urea	0.21g/L	0.19-0.45 g/L

The association of vitamin D deficiency with elevated parathyroid hormone (PTH) levels suggests secondary hyperparathyroidism, which may have contributed to increased bone fragility in this patient. Based on the clinical findings, imaging, low bone mineral density, and family history, the presentation was highly suggestive of type I OI, although no genetic testing was performed. Vitamin D supplementation was initiated prior to intravenous zoledronic acid at a dose of 5 mg annually.

Informed consent

Written informed consent was obtained from the patient for the publication of this case report and the accompanying images.

## Discussion

OI is an inherited connective tissue disorder caused by abnormalities in the synthesis or structure of type I collagen. Most cases result from autosomal dominant mutations in the *COL1A1* or *COL1A2* genes, although recessive forms have also been described [1,2]. Non-lethal forms (types I and IV according to the Sillence classification) typically present with recurrent low-energy fractures, blue sclerae, and short stature [2,3].

Recurrent fractures following minimal trauma represent a key diagnostic feature of OI and may lead to progressive bone deformities and joint instability [3]. In our case, fractures began at the age of eight and persisted throughout adolescence, which is consistent with moderate forms of OI [4].

Bone fragility in OI is not solely explained by decreased bone mineral density (BMD), but also by qualitative abnormalities of type I collagen, the main component of the bone matrix. These alterations impair bone strength and predispose to fractures, even in the presence of relatively preserved BMD [2,5,6]. In our patient, the fracture burden appeared disproportionate to densitometric findings, supporting a predominant qualitative defect of the bone matrix.

Blue sclerae remain a classic extra-skeletal feature of OI and result from thinning of scleral collagen, allowing visualization of the underlying choroidal vasculature [5,7]. Its presence in our patient supports a mild to moderate phenotype [1,3]. Short stature, also observed in this case, is frequently reported in moderate forms and may result from recurrent fractures, vertebral compression, and skeletal deformities [5,8].

The long-bone deformities observed in this case are likely due to repeated fractures with inadequate consolidation, a pattern commonly described in untreated or late-treated patients [3,5]. Unlike severe forms (type III), which are often associated with early deformities and respiratory complications [1,9,10], our patient did not exhibit hearing impairment, dentinogenesis imperfecta, or respiratory insufficiency, supporting a non-lethal phenotype.

Radiographic evaluation is central to the assessment and typically demonstrates diffuse bone demineralization, cortical thinning, fractures, and long-bone deformities [3]. In our case, imaging and dual-energy X-ray absorptiometry (DXA) findings confirmed severe bone loss and deformities, consistent with previously reported data.

In addition, the association of vitamin D deficiency with elevated parathyroid hormone levels suggests secondary hyperparathyroidism, which may have contributed to increased bone fragility and fracture risk in this patient. This highlights the importance of identifying and correcting modifiable metabolic factors that may exacerbate skeletal fragility [2].

Differential diagnoses of recurrent fractures in young individuals include metabolic bone diseases such as rickets, hypophosphatasia, and other skeletal dysplasias [2,3]. However, the combination of recurrent low-energy fractures, blue sclerae, and a positive family history strongly favors a diagnosis within the spectrum of OI.

According to the literature, bisphosphonates are the cornerstone of treatment in OI and have been shown to improve bone mineral density and reduce fracture rates, particularly in children and adolescents [11,12]. In adults with milder phenotypes, teriparatide has also demonstrated beneficial effects on bone density [13]. In our patient, the absence of early treatment may have contributed to the progression of skeletal deformities. Given persistent bone fragility during transition to adulthood, initiation of intravenous bisphosphonate therapy was appropriate.

Recent studies emphasize the importance of structured follow-up into adulthood [14]. At 19 years of age, our patient is in this transitional phase, warranting continued multidisciplinary management to prevent long-term complications [15].

Limitations

A major limitation of this case is the absence of genetic testing, which prevents molecular confirmation of the diagnosis. Although osteogenesis imperfecta is most commonly associated with mutations in the *COL1A1* and *COL1A2* genes, such investigations were not available in our setting due to financial constraints. Consequently, the diagnosis remains clinically based and cannot be considered definitive. Nevertheless, the combination of characteristic clinical features, a positive family history, and typical radiological findings strongly supports a diagnosis within the spectrum of osteogenesis imperfecta. Similar approaches have been reported in the literature, where the diagnosis relies on clinical and radiological findings, particularly in resource-limited settings where genetic testing is not readily accessible [5].

## Conclusions

This case highlights the importance of recognizing the classical clinical features of osteogenesis imperfecta to avoid diagnostic delay. It also underscores the challenges of diagnosing mild forms in adulthood, particularly when early signs are overlooked. While clinical and radiological findings may strongly suggest the diagnosis, genetic testing remains the gold standard for confirmation and appropriate classification. Early recognition is essential to prevent complications and to ensure appropriate multidisciplinary management and genetic counseling when available.
